# Mental health distress and associated factors among online CBT service users during the Iron Swords War

**DOI:** 10.3389/fpsyg.2026.1788592

**Published:** 2026-04-13

**Authors:** G. Hirsh-Yechezkel, I. Zaslavsky-Paltiel, A. Farhi, S. Edelman-Yaron, R. Sarafraz, G. Barkai, H. Amir, J. Porat

**Affiliations:** 1 Gertner Institute for Epidemiology and Health Policy Research, Sheba Medical Center, Tel Hashomer, Ramat Gan, Israel; 2 Sheba Beyond Virtual Hospital, Sheba Medical Center, Tel Hashomer, Ramat Gan, Israel; 3 Gray Faculty of Medical & Health Sciences, Tel-Aviv University, Tel-Aviv, Israel

**Keywords:** anxiety, depression, online-CBT, PTSD, war

## Abstract

**Background:**

The October 7, 2023 attacks in Israel and the ensuing Iron Swords War triggered a widespread mental health crisis. In this context, online cognitive behavioral therapy (CBT) has emerged as an accessible and convenient option.

**Objective:**

To characterize individuals who sought out online CBT during the war, with regard to their sociodemographic characteristics, wartime exposures, and self-reported psychological distress and to evaluate the associations between sub-populations characteristic and psychological distress severity.

**Methods:**

Data were analyzed from electronic medical records of civilians who contacted Sheba BEYOND’s virtual CBT clinic between November 2023 and August 2024. Sociodemographic characteristics, psychiatric history, and wartime exposures were available for 520 adults, 199 of whom also completed standardized psychological questionnaires (including PDS-5, GAD-7, PHQ-9) which were used to measure sever psychological symptoms of PTSD, anxiety and depression.

**Results:**

Individuals seeking online services during the war represented a diverse population in terms of their socio-demographic background (e.g., age range; level of educational, employment status, and place of residence) and war-related experiences. High rates of psychological symptoms were observed: 62.3% of respondents scored ≥36 on the PDS-5, 43.2% met criteria for severe anxiety (GAD-7 score ≥15), and 20.1% met criteria for severe depression (PHQ-9 score ≥20). The multivariable model indicated that displaced individuals were at a significantly elevated risk for PTSD (OR = 6.33; 95% CI 1.74–23.05), for anxiety symptoms (OR = 4.67; 95% CI 1.76–12.41), and for depressive symptoms (OR = 2.97; 95% CI 1.05–8.36). In addition, history of interpersonal trauma was associated with increase the risk of severe PTSD, anxiety, and depressive symptoms.

**Discussion:**

The high level of psychological distress observed in this study highlight the importance of accessible wartime mental health care. By reaching populations across diverse sociodemographic backgrounds and varying levels of trauma exposure, online interventions demonstrate the potential feasibility and accessibility necessary to provide mental health support during time of crisis. The identification of both high symptom severity rates and subpopulation at higher risk provides valuable information for future preparedness for such services during emergencies.

## Background

The October 7, 2023 Hamas attacks on Israel and the ensuing Iron Swords War triggered a widespread mental health crisis among the Israeli population. Recent studies have highlighted a substantial increase in post-traumatic stress symptoms (PTSD), anxiety, and depression among civilians. This is especially evident among people directly exposed to trauma, including residents of southern Israel, soldiers, and individuals who experienced traumatic loss (Levi-Belz et al., 2024, 2025; Amsalem et al., 2025; Neria et al., 2025). Displaced populations have also been identified as particularly vulnerable, possibly due to both the acute trauma as well as the cumulative burden of displacement (Marciano et al., 2024; Amsalem et al., 2025; Hamama et al., 2025; Shapira et al., 2025).

According to a study conducted on a sample of the Israeli population, the rate of probable PTSD increased from 16.2% before the war to 29.8% 1 month after its onset. Symptoms of anxiety and depression also rose, from 24.9% to 42.7% and from 31.3% to 44.8%, respectively (Levi-Belz et al., 2024). Individuals directly exposed to trauma during the initial October 2023 attack continued to exhibit elevated rates of PTSD (40.3%) and depression (45.2%) 5 months after the attack (Levi-Belz et al., 2025). A survey conducted on May 2024 demonstrated that 24 and 31% of residents in conflict-affected areas reported elevated anxiety and depressive symptoms respectively, and 37% screened above the clinical threshold for posttraumatic stress symptoms (Amsalem et al., 2025).

Studies from previous wars in Israel, as well as from other conflict-affected regions worldwide, have also reported elevated levels of mental distress (Greene et al., 2018). For example, during Operation Cast Lead, Israeli young adults near the Gaza Strip showed high rates of PTSD (20.0%), anxiety (57.8%), and depression (45.2%), which significantly decreased months after its end. Another study found that shorter proximity to the Gaza Strip, defined by the time available to seek shelter, correlated with higher symptom rates, with those having only 15 s to shelter showing the highest PTSD and depression levels (Bar-Haim et al., 2010; Neria et al., 2010). A global meta-analysis found average prevalence rates of PTSD, anxiety, and depression at 23.5%, 30.7%, and 28.9%, respectively, with significant differences between wartime and post-war periods. Vulnerable groups, such as displaced persons, showed particularly high distress; for instance, a 2022 study in Ukraine found increased PTSD symptoms among displaced individuals, and in southern Ethiopia, PTSD prevalence among internally displaced persons reached 58.4% (Madoro et al., 2020; Ben-Ezra et al., 2023).

Given the sharp rise in psychological distress during conflict situations, there was an increasing and urgent need for accessible mental health services. However, despite efforts to expand psychological care during the Iron Swords War, only a small fraction of the affected population have received treatment (State Comptroller and Ombudsman of Israel, 2024; Neria et al., 2025).

In Israel, mental health care is provided mainly through HMOs and hospitals, which were already under strain due to workforce shortages and long waiting times (State Comptroller and Ombudsman of Israel, 2024; Krivoy and Rosenthal, 2025). Following the attack, demand for mental health services dramatically surged, as reflected in a 46% increase in emergency psychiatric visits (Shalev et al., 2024), a rise in crisis helpline contacts (Daniels et al., 2026) and a 10-fold increase in first-time benzodiazepine prescriptions (Rahamim et al., 2025). In response, several initiatives were implemented, including trauma clinics, resilience centers, resilience coaches, and the involvement of primary care physicians (Beri Drezner et al., 2024; Rahamim et al., 2025; Reuveni et al., 2025; Lurie et al., 2026). In addition, the National Insurance Institute’s anxiety protocol for Victims of Hostilities was activated, making mental health services accessible through publicly funded programs (National Insurance Institute of Israel, 2025).

In such circumstances, online mental health interventions have become an important solution to bridge this treatment gap, offering accessible and convenient options for managing distress during crises (Peterson et al., 2022; Zandieh et al., 2024; Smulson et al., 2025). Online therapy reduces logistical barriers, shortens wait times, lowers costs, and helps maintain continuous care during service disruptions (Casey et al., 2014; Ferracioli et al., 2023). These benefits make online interventions especially important when in-person care is restricted such as during war or national emergencies (Chorna, 2024; Sberro-Cohen and E Ellen, 2025; Smulson et al., 2025).

Within days of the October 7th attack, a dedicated clinic was established at Sheba BEYOND, Sheba Medical Center’s virtual hospital. The clinic was created in response to the rising demand for mental health services and as a solution to wartime related challenges to accessibility and availability of care. The clinic was open to self-referral and treatment was funded by the National Insurance Institute, enabling free of charge for civilians across the country from the comfort and safety of their own homes. The clinic offers therapist-guided online treatment, delivered via videoconferencing (e.g., Zoom). Treatment is based on cognitive-behavioral therapy (CBT) using evidence-based protocols for adult civilians experiencing anxiety depression or PTSD symptoms. Virtual treatment has been shown to be as effective as face-to-face CBT (Casey et al., 2014; Sijbrandij et al., 2016; Matsumoto et al., 2021).

The current study aimed to characterize individuals who sought psychological services during the Iron Swords War at the clinic, with respect to their pre-treatment sociodemographic profiles, wartime circumstances (including traumatic events) and self-reported psychological distress, as well as to assess the associations between population characteristics and psychological distress severity. Additionally, the study describe the change in psychological test scores post-treatment among a sample of the population who completed post treatment assessment.

## Methods

This study analyzed data from adult individuals who contacted Sheba BEYOND’s virtual psychology clinic of Sheba Medical Center, a major tertiary medical center in Israel. The virtual psychology clinic provides remote CBT-based treatment free-of-charge to self-referred civilians with war-related symptoms of anxiety, depression and PTSD. Clinic attendees underwent a comprehensive intake to establish diagnosis and treatment plan. Patients who met diagnostic criteria for PTSD were referred to an Cognitive Processing Therapy (CPT) treatment track, while patients with anxiety, mild-to-moderate depression, or other war-related distress received about 12-session CBT course tailored to their needs. All treatments were delivered in one-to-one sessions. At initial contact, as part of routine clinical intake, individuals completed a digital intake survey collecting sociodemographic information, psychiatric history (diagnoses and treatments), and information on traumatic experiences endured by the respondent in relation to the October 7th attack and its subsequent consequences. Soldiers were excluded from the study because they were routinely referred to dedicated military mental health clinics. Individuals with a history of psychiatric hospitalization were excluded because they were typically referred to psychiatric clinics, as once-weekly online treatment was not considered an appropriate treatment modality for major psychiatric conditions. The study included 520 participants who contacted the clinic between November 2023 and August 2024 and completed the intake survey, of whom 199 opted to complete standardized psychological questionnaires.

No significant differences (*p* > 0.05) were found between those who opted to completed the psychological questionnaires (*n* = 199) and those who did not (*n* = 321) across all sociodemographic and war-related variables listed in Table 1.

**Table 1:** Sociodemographic characteristics of study population.

Variable	*N* = 520	100.0%*
Age		
18–29	154	29.6
30–39	131	25.2
40–49	98	18.9
50–59	70	13.5
60+	67	12.9
Gender		
Male	153	29.4
Female	367	70.6
Country of birth		
Israel	435	83.7
Not-Israel	85	16.3
Education		
Primary/ secondary/high school	172	33.1
Post high school non-academic	114	21.9
Bachelor’s degree or higher	234	45.0
Employment status		
Salaried employee	276	53.1
Self-employed	64	12.3
Unemployed (including receiving disability benefits)	117	22.5
Retired	44	8.5
National service or studies	19	3.7
Socio-Economic Index		
1–3 1st tertile	37	7.4
4–6 2nd tertile	143	28.7
7–10 3rd tertile	318	63.9
Peripherality Index		
1–4 1st tertile	105	21.1
5–7 2nd tertile	134	26.9
8–10 3rd tertile	259	52.0
Past psychiatric diagnosis**		
Yes	207	39.8
No	313	60.2
Displacement status (DS)		
Permanent home	432	84.7
Evacuated	78	15.3
Time to shelter (TTS)		
0–15 min	73	14.0
30–60 min	108	20.8
90 min	339	65.2
War-related exposure (WRE)		
High	289	55.6
Moderate	175	33.7
Unknown	56	10.8

*For 22 cases, peripheral and socioeconomic cluster information was missing; for 10 cases, displacement status was unknown. Percentages were calculated excluding missing values.

**Self-reported history of psychiatric diagnosis or use of psychiatric medication.

In addition to basic sociodemographic variables (age, gender etc.), the analysis included two area-level indices based on the Israeli Central Bureau of Statistics (ICBS) classification: the Peripherality Index and the Sociodemographic Index. These indices rank localities according to geographic proximity to resources and socioeconomic characteristics, respectively, and classify them into 10 clusters. For this study, the clusters were grouped into low, medium, and high categories based on tertiles from the 2019 to 2020 national distribution (Israel Central Bureau of Statistics, 2023a No. 1903; Israel Central Bureau of Statistics, 2023b No. 1917).

Four variables collected in the intake survey were utilized to characterize participants’ status during the war. Displacement status (DS) was based on self-reported evacuation from a permanent residence. This included authority-mandated evacuation as well as self-evacuation due to proximity to conflict zones or inadequate shelter conditions. Time to Shelter (TTS) was based on the Home Front Command’s official directive (Israel Home Front Command, 2025) specifying the recommended timeframe during rocket alerts to reach shelter. Border localities were assigned a TTS of 0 s, while in central Israel this time extended up to 90 s. The variable TTS served as an indication of threat intensity. War-Related Exposure (WRE) referred to participants’ reports of traumatic experience they endured in relation to the October 7 attacks and the ongoing war, selected from a predefined list. Events were classified into two levels: High exposure included being present at the attacked music festival, located within close proximity to terrorist infiltration, having a friend or family member who has been injured, killed or kidnapped, exposure to combat as a member of the military or rapid-response team, having a close family member in active combat service, or sustaining a personal injury. Low exposure included for example media exposure (including stress related to air raid sirens), significant disruptions to daily functioning or occupation, and non-combat volunteer activities. Participants were allowed to select multiple exposures and were included in the high exposure group if they experienced at least one event rated as high exposure.

Three psychological conditions were evaluated upon intake into the service by validated psychological tests. The PDS-5 is a self-report questionnaire with 20 items assessing PTSD symptoms, based on DSM-5 criteria. Symptoms are rated on a 5-point Likert scale ranging from 0 (not at all) to 4 (6 or more times a week), with total scores ranging from 0 to 80. Based on established cutoffs, total scores of 0–17 indicate no clinically significant PTSD symptoms, 18–27 indicate partial PTSD, while a cutoff of 28 is commonly used to indicate probable PTSD (Foa et al., 2016). Due to the high proportion of participants exceeding this threshold in our sample, analyses used a more conservative cutoff of 36, (reported sensitivity = 65%, specificity = 84%) (Wittmann et al., 2021). The GAD-7 is a 7-item self-report scale designed to assess symptoms of generalized anxiety. Each item is rated on a 4-point Likert scale ranging from 0 (“not at all”) to 3 (“nearly every day”), resulting in a total score between 0 and 21. A cut-off score of 10 or above has been shown to yield high sensitivity and specificity (both exceeding 0.80) for probable cases of GAD. However, a higher cut-off score of 15 was selected in the present study, given the elevated prevalence of anxiety symptoms during the war (Spitzer et al., 2006). The PHQ-9 is a 9-item self-report measure assessing depressive symptoms. Items are rated on a 0–3 Likert scale, with total scores ranging from 0 to 27. A score of 20 or higher was used to indicate severe depressive symptoms, representing high validity category on the PHQ-9 (Kroenke et al., 2001).

Internal consistency (Cronbach’s *α*) for the psychological questionnaires in the current sample were 0.92 for the PDS-5, 0.87 for the GAD-7, and 0.83 for the PHQ-9.

Past trauma exposure (PTE), predating the war, was assessed using a predefined list of traumatic events. Participants could select multiple types that were categorized into three exclusive groups based on a hierarchy: interpersonal trauma (child abuse, physical or sexual assault), non-interpersonal trauma (military/combat events, accidents, natural disasters, serious illness), or no reported trauma. Participants were categorized into one trauma group only, with interpersonal trauma taking priority when multiple trauma types were reported.

### Statistical analysis

Descriptive statistics were used to summarize the sociodemographic and war-related characteristics of the total study population (520 participants), presented as absolute frequencies and percentages. Psychological outcomes were analyzed in a subsample of participants who had completed standardized self-report questionnaires (*n* = 199), using predefined thresholds: PDS-5 ≥ 36, GAD-7 ≥ 15, and PHQ-9 ≥ 20 to indicate severe symptom levels.

Univariate analyses were conducted to assess associations between sociodemographic or war-related variables and each psychological outcome. Chi-square tests were applied to compare participants scoring above and below each threshold. Variables that were significantly associated with outcomes in the univariate analyses were included in multivariable logistic regression models to estimate adjusted odds ratios (ORs) with 95% confidence intervals (CIs) for severe symptomatology.

Additionally, to assess potential changes in symptom severity during treatment, paired t-tests were conducted on total scores among participants who completed both intake and post-treatment questionnaires, provided their baseline scores exceeded the subclinical threshold. Effect sizes for mean score change were calculated using Cohen’s d for paired samples, defined as the mean difference divided by the standard deviation of the differences.

Data were analyzed using SAS software, version 9.4 (SAS Institute, Cary, NC, USA). The study was approved by the Institutional Review Board (IRB) of Sheba Medical Center.

## Results

**Figure 1:**
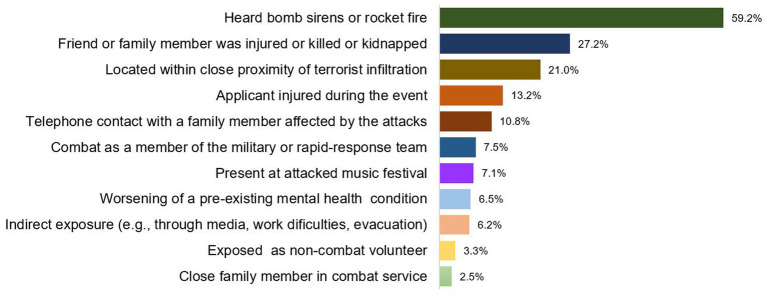
Type of war related exposure (WRE).

Table 1 presents the sociodemographic characteristics and war-related variables of the total study population (*n* = 520) and Figure 1 presents the types of WRE reported by participants in relation to the October 7th attacks and the subsequent war period.

More than half of the population were under the age of 40 29.6% were aged 18–29, and an additional 25.2% were aged 30–39. Nevertheless, 12.9% of those who contacted the service were aged 60 or older. The majority of participants (70.6%) were women, and 83.7% were Israeli-born. Approximately one-third of participants reported a primary to high school education, while 45.0% held an academic degree. In terms of employment status, 53.1% were salaried employees, and 31.0% were unemployed or retired. A total of 63.9% of participants were classified in the highest national socioeconomic tertile, corresponding to clusters 7–10 of the Israeli Central Bureau of Statistics’ socioeconomic index, while 7.4% were classified in the lowest tertile (clusters 1–3). Additionally, 52.0% live in areas considered non-peripheral, corresponding to the top tertile (clusters 8–10) of the national geographic peripherality index, 21.1% were in the lowest tertile, indicating high peripherality. Almost 40% of the population had history of psychiatric diagnosis.

While most participants remained in their permanent homes, 11.6% were evacuated under government-sponsored evacuation programs and an additional 3.7% left their homes independently due to missile threats (primarily from the north or south) or other war-related reasons. 14.0% of participants were found to have originally resided in areas with the shortest TTS (0–15 s), indicating a high risk of exposure.

According to Figure 1, nearly 60% of respondents identified sirens or rocket fire as a traumatic event. Additionally, 27.2% reported having a close family member or friend who was injured, killed, or kidnapped during the events. About 21% were located within close proximity to the terrorist infiltration during the October 7th attacks, and 13.2% of respondents reported being physically injured during the events. Furthermore, 10.8% reported telephone contact with a family member affected by the attacks while they were ongoing and described this experience as traumatic.

A total of 199 participants completed three psychological screening questionnaires at the time of their initial contact with the service: PDS-5, GAD-7 and PHQ-9. No significant differences were found in the distribution of demographic variables between 199 participants who completed the questionnaire and 321 who did not (data not shown).

Table 2 presents the mean scores and the frequency distributions across severity categories for the three psychological screening measures.

**Table 2:** Distribution of PDS-5, GAD-7 and PHQ-9 test scores*.

Score	Category	*N* = 199	100.0%	Mean ± SD
PDS-5 posttraumatic diagnostic scale				41.1 ± 16.3
0–17	None	15	7.5	
18–27	Partial PTSD	28	14.1	
28–80	Full PTSD	156	78.4	
GAD-7 generalized anxiety disorder				12.8 ± 5.1
0–4	Minimal anxiety	17	8.5	
5–9	Mild anxiety	34	17.1	
10–14	Moderate anxiety	62	31.2	
15–21	Severe anxiety	86	43.2	
PHQ-9 patient health questionnaire				14.6 ± 5.9
0–4	No depression	11	5.5	
5–9	Mild depression	34	17.1	
10–14	Moderate depression	46	23.1	
15–19	Moderate to severe depression	68	34.2	
20–27	Severe depression	40	20.1	

*Test scores reflect assessments conducted before the initial referral to psychological services.

The PDS-5, the mean score was 41.1 (±16.3). The vast majority of respondents (78.4%) met the criteria for full PTSD (score ≥ 28), 14.1% met criteria for partial PTSD (scores 18–27), and only 7.5% fell below the clinical threshold (score < 18). Since nearly 80% of the respondents scored above 28, a more stringent threshold of 36 was adopted for subsequent analyses (Wittmann et al., 2021). 62.3% of the respondents met the modified threshold for elevated PTSD symptom severity. The mean score for GAD-7 was 12.8 (±5.1). Severe anxiety (score ≥ 15) was reported by 43.2% of participants, and 31.2% had moderate anxiety. In addition, 17.1% had mild anxiety, and 8.5% had minimal anxiety (score < 5). Regarding depressive symptoms measured by the PHQ-9, the mean score was 14.6 (±5.9). 20.1% met criteria for severe depression (score ≥ 20), about third of the sample (34.2%) reported moderate-to-severe depression (scores 15–19), while only 5.5% and 17.1% reported no or low symptoms, respectively.

Regarding PTEs, 25.6% reported military or combat-related trauma in the past, 24.6% experienced trauma related to serious illness, and approximately 20% reported childhood abuse, car accidents, and/or sexual abuse. Physical abuse was reported by 13.6% of participants, while 40.2% did not report any past trauma (Table 3).

**Table 3:** Past trauma exposure (PTE).

Past trauma exposures	*n*	%
Military or combat-related	51	25.6
Child abuse	42	21.1
Accident	40	20.1
Natural disaster	9	4.5
Serious, life-threatening illness	49	24.6
Physical assault	27	13.6
Sexual assault	42	21.1
Past trauma categories	*N* = 199	100.0%
Interpersonal trauma**	75	37.7
non-interpersonal trauma***	44	22.1
None	80	40.2

*Each individual could select multiple traumas, so the percentage refers to the percentage of respondents who chose this trauma, regardless of their other selections.

**Including child abuse and physical assault and sexual assault.

***Including military or combat-related exposure, accident, natural disaster and serious, life-threatening illness.

Table 4 present a univariate association between socioeconomic or war-related factors and psychological symptom severity. Displaced individuals showed higher prevalence of symptoms: 88.0% scored PDS-5 ≥ 36, compared to 58.6% of non-displaced (*p* = 0.005), 68.0% score GAD-7 ≥ 15 compared to 39.7% (*p* = 0.008) and 32.0% scored PHQ-9 ≥ 20 compared to 18.4% (*p* = 0.11). Individuals with a history of psychiatric diagnosis also exhibited higher risk for distress symptoms compared with people who did not report psychological diagnosis (70.6% vs. 56.1% *p* = 0.04, 47.1% vs. 40.4% *p* = 0.3 and 28.2% vs. 14.0% *p* = 0.01 for PDS-5 ≥ 36, GAD-7 ≥ 15 and PHQ-9 ≥ 20, respectively). Other war-related circumstances were not found to have significantly association with the test scores. In addition, a trend can be observed across the three groups of PTE with regard to the rate of severe PTSD (PDS-5 ≥ 36): starting with those who experienced interpersonal trauma (77.3%), followed by those who experienced other trauma (62.8%), and ending with those who reported no past trauma (49.4%) (*p* = 0.002). A similar decreasing trend was also observed for anxiety and depression (61.3%, 41.9%, 27.9% and 33.3%, 18.6%, 8.9% for GAD-7 ≥ 15 and PHQ-9 ≥ 20, respectively).

**Table 4:** Variable associated with highest score in the psychological test - univariate model.

		PDS5 ≥ 36			GAD7 ≥ 15			PHQ9 ≥ 20		
Variables	N	*n*	%*	*p* value	*n*	%*	*p* value	*n*	%*	*p* value
Total	199	124	62.3		86	43.2		40	20.1	
Age				0.5			0.6			0.8
18–45	128	82	64.1		57	44.5		25	19.5	
45+	71	42	59.2		29	40.9		15	21.1	
Sex				0.4			0.2			1.0
Male	49	28	57.1		17	34.7		10	20.4	
Female	150	96	64.0		69	46.0		30	20.0	
Education				0.4			0.1			0.5
Primary/secondary/high school	59	39	66.1		32	54.2		14	23.7	
Post high school non-academic	43	23	53.5		15	34.9		6	14.0	
Academic	97	62	63.9		39	40.2		20	20.6	
Country of birth				0.8			0.8			0.6
Israel	163	101	62.0		71	43.6		34	20.9	
Not-Israel	36	23	63.9		15	41.7		6	16.7	
Employment status				0.14			0.4			0.2
Salaried employee	109	64	58.7		43	39.5		17	15.6	
Self-employed	31	18	58.1		12	38.7		6	19.4	
Unemployed **	41	32	78.1		22	53.7		12	29.3	
Other	18	10	55.6		9	50.0		5	27.8	
Displacement status				0.005			0.008			0.11
Permanent home	174	102	58.6		69	39.7		32	18.4	
Displaced	25	22	88.0		17	68.0		8	32.0	
Time since event				0.4			0.6			0.5
<30 days	46	26	56.5		21	45.7		7	15.2	
>30	139	89	64.0		57	41.0		28	20.1	
Past psychiatric diagnosis***				0.04			0.3			0.01
Yes	85	60	70.6		40	47.1		24	28.2	
No	114	64	56.1		46	40.4		16	14.0	
Peripherality cluster				0.9			0.8			0.8
1–4 1st tertile	40	26	65.0		19	47.5		7	17.5	
5–7 2nd tertile	53	32	60.4		24	45.3		11	20.8	
8–10 3rd tertile	98	63	64.3		41	41.8		22	22.5	
SES cluster				0.5			0.3			0.2
1–3 1st tertile	18	12	66.7		6	33.3		2	11.1	
4–6 2nd tertile	61	42	68.9		31	50.8		17	27.9	
7–10 3rd tertile	112	67	59.8		47	42.0		21	18.8	
Time to shelter (TTS)				0.16			0.6			0.8
0–15 min	26	19	73.1		13	50.0		4	15.4	
30–60 min	43	22	51.2		20	46.5		9	20.9	
90 min	130	83	63.9		53	40.8		27	20.8	
War related exposure (WRE)				0.6			0.7			0.9
Highly WRE	115	74	64.4		49	42.6		23	20.0	
Moderate WRE	82	50	61.0		37	45.1		17	20.7	
Past trauma exposure (PTE)****				0.002			<0.001			<0.001
Interpersonal trauma	75	58	77.3		46	61.3		25	33.3	
Non-interpersonal trauma	43	27	62.8		18	41.9		8	18.6	
None	79	39	49.4		22	27.9		7	8.9	

*Percent (%) within each category; Percentages were calculated excluding missing values; For 8 cases, peripheral and socioeconomic cluster information was missing; for 14 cases TTS could not be determined; for 2 people WRE were missing.

**including individuals receiving disability benefits and those retired.

***Self-reported history of psychiatric diagnosis or use of psychiatric medication.

Results of the multivariable model (Table 5) indicated that individuals who were displaced were at a significantly elevated risk for PTSD (OR = 6.33; 95% CI 1.74–23.05 *p* = 0.005), for anxiety symptoms (OR = 4.67; 95% CI 1.76–12.41 *p* = 0.002), and for depressive symptoms (OR = 2.97; 95% CI 1.05–8.36 *p* = 0.04). Furthermore, individuals with a history of interpersonal trauma showed significantly higher levels of PTSD, anxiety and depressive symptoms compared to those who reported no past exposure to trauma (OR = 3.58; 95% CI 1.70–7.57 *p* < 0.001, OR = 5.25; 95% CI 2.46–11.19 *p* < 0.001, and OR = 4.77; 95% CI 1.81–12.60 *p* = 0.002, respectively).

**Table 5:** Factors associated with symptom severity - multivariate model.

Variable	PDS-5 ≥ 36		GAD-7 ≥ 15		PHQ-9 ≥ 20	
	OR	95%CI	OR	95%CI	OR	95%CI
Age						
18–45	1.00		1.00		1.00	
45+	0.92	0.47–1.79	0.92	0.47–1.80	1.27	0.56–2.84
Gender						
Male	1.00		1.00		1.00	
Female	1.13	0.54–2.35	1.52	0.72–3.19	0.86	0.36–2.05
Psychiatric diagnosis						
No	1.00		1.00		1.00	
Yes	1.38	0.71–2.67	0.80	0.41–1.54	1.72	0.79–3.76
Displacement status						
Permanent home	1.00		1.00		1.00	
Displaced	6.33	1.74–23.05	4.67	1.76–12.41	2.97	1.05–8.36
War related exposure (WRE)						
Moderate WRE	1.00		1.00		1.00	
High WRE	1.01	0.53–1.93	0.74	0.39–1.41	0.99	0.45–2.20
Past trauma exposure (PTE)						
None	1.00		1.00		1.00	
No-interpersonal	1.66	0.72–3.82	2.00	0.85–4.74	1.83	0.57–5.86
Interpersonal	3.58	1.70–7.57	5.25	2.46–11.19	4.77	1.81–12.60

Finally, analyses were conducted on a subsample of 38 cases for which both intake and post-treatment questionnaire data were available, provided their baseline scores exceeded the subclinical threshold. The mean time interval between the two assessments was 3.5 (SD = 1.4) months, and the mean number of treatment sessions was 12.5 (SD = 5.4). A reduction in mean symptom scores was observed across all three measures. Specifically, PDS-5 scores were reduced from 45.1 to 28.2, GAD-7 scores from 14.7 to 7.6, and PHQ-9 scores from 15.9 to 9.9 (all *p* < 0.001). Cohen’s d values for mean score changes were 0.95 for PDS-5, 1.06 for GAD-7, and 0.73 for PHQ-9, indicating notable changes in this subsample.

## Discussion

This study characterizes the population that sought out a virtual cognitive behavioral therapy (CBT) clinic that provided counseling to a wide range of populations in Israel during the Iron Swords War and assesses the severity of psychological distress of these patients during the first 10 months of the war. The findings show that this service was accessed by a socio-demographically and geographically diverse population. The sample encompassed a broad age range and included participants across diverse levels of educational attainment and types of employment. Certain subgroups were represented at higher rates; for example, women, who have been shown to report higher levels of psychological distress and to utilize mental health services more frequently (Lurie et al., 2026; State Comptroller and Ombudsman of Israel, 2024). In addition, most of the participants presented with severe mental health symptoms. Overall 78.4%, 43.2%, and 20.1% reported symptoms consistent with severe PTSD, anxiety and depression, respectively. Two subgroups were identified at elevated risk for PTSD anxiety, and depression symptoms: those displaced from their homes and those with a history of interpersonal trauma (such as sexual or physical assault or childhood abuse). Compared to non-displaced individuals, displaced individuals had an approximately sixfold prevalence of severe PTSD symptoms, nearly fivefold prevalence of anxiety symptoms, and around threefold prevalence of depression symptoms. Past interpersonal trauma was associated with a 3.6-fold increase in risk for severe PTSD symptoms, and an approximately fivefold increase for anxiety or depression compared to individuals with no history of such trauma.

The rate of PTSD in the study population was higher than that reported in other Israeli cohorts during the Iron Swords War (Levi-Belz et al., 2024; Amsalem et al., 2025). Our sample is comprised of individuals actively seeking psychological treatment during the war and therefore possibly at higher risk than population based samples, as shown in past studies that specifically assessed populations who independently sought mental health services (Dominguez et al., 2004; Sheerin et al., 2016; Adams et al., 2017; da Silva et al., 2019; Lu et al., 2021). However, the rate of severe anxiety and depression were not higher than those reported in other studies (Peleg and Gendelman, 2025). It should be note that elevated scores on screening tests for PTSD or other mental illness indicate symptom severity alone, and do not necessarily equate to formal clinical diagnosis (Foa et al., 2016). Consistent with this, a predictive modeling of PTSD prevalence in Israel during the war estimates that only 5.3% of the general population may develop clinical PTSD (Katsoty et al., 2024), much lower than the rate of severe PTSD symptoms reported in surveys (Peleg and Gendelman, 2025). However, the use of screening tools remains highly valuable for assessing the mental health status of the population during wartime. Moreover, data collected during the first months after the traumatic event may have captured peak levels of distress, including Acute Stress Disorder (ASD). Although ASD is a well-recognized risk factor for the development of PTSD, not all individuals with ASD progress to chronic symptoms (Bonanno et al., 2011; Bryant et al., 2011). Nevertheless early trauma-focused treatment for those meeting ASD criteria has been shown to be important as it accelerate recovery and reduce the transition to chronic PTSD (Bryant et al., 2011; Roberts et al., 2019).

Interestingly, contrary to expectations based on other studies in Israel (Amsalem et al., 2025; Levi-Belz et al., 2025; Peleg and Gendelman, 2025), the results demonstrate high mental distress scores among civilian residents of central Israel, with no significant differences in test scores compared to individuals living in the high-conflict zones. It should be noted that there were multiple fronts during the war, placing the entire country effectively under constant threat. Residents in central Israel were also exposed to trauma, primarily indirect, including repeated alerts of incoming missile attacks as well as missile landings in their areas of residence. Many, especially younger individuals, had close friends or family members serving in the army, and some served themselves in combat zones. In addition, the nationwide exposure to horrifying news reports, including live broadcasts and images, evoked immense fear among the population and constituted a traumatic experience in itself (Bitton et al., 2017; Zhu et al., 2023; Kaim and Bodas, 2024; Zerach, 2024). These events may explain psychological distress even in areas far from the conflict zones, particularly among individuals who suffered and sought treatment.

The current study has several limitations. The study population consists of individuals who voluntarily sought psychological services and therefore may not represent the entire population in need of treatment, nor the general population in Israel. Additionally, since the service is provided online, the study population was composed mainly of individuals with access to suitable technology and who likely have higher digital literacy than the general population. In addition, lack of comparison group such as individual receiving face-to-face treatment or non-help-seeking civilians limits interpretation of the findings.

In summary, the study’s findings provide a better understanding of psychological distress reported by individuals seeking treatment during the war and their need for accessible psychological treatment. The data demonstrate that online CBT is able to reach individuals from diverse geographic regions, sociodemographic backgrounds, and trauma intensity levels. Furthermore, the identification of both high symptom severity and subpopulations at elevated risk provides important insights for future preparedness of mental health services, including the type and intensity treatment needed, as well as allocation of support during emergencies.

## Data Availability

The datasets presented in this article are not readily available because of the sensitive nature of the study. We can share non identifiable data upon request. Access may be limited to specific variables that cannot be used to identify small groups, and is subject to approval of the ethical committee. Requests to access the datasets should be directed to the corresponding author.

## References

[ref1] Adams R. E. Urosevich T. G. Hoffman S. N. Kirchner H. L. Hyacinthe J. C. Figley C. R. . (2017). Social support, help-seeking, and mental health outcomes among veterans in non-VA facilities: results from the veterans’ health study. Mil. Behav. Health 5, 393–405. doi: 10.1080/21635781.2017.1333067, 29098116 PMC5663244

[ref2] Amsalem D. Haim-Nachum S. Lazarov A. Levi-Belz Y. Markowitz J. C. Bergman M. . (2025). The effects of war-related experiences on mental health symptoms of individuals living in conflict zones: a longitudinal study. Sci. Rep. 15:889. doi: 10.1038/S41598-024-84410-3, 39762464 PMC11704351

[ref3] Bar-Haim Y. Holoshitz Y. Eldar S. Frenkel T. I. Muller D. Charney D. S. . (2010). Life-threatening danger and suppression of attention bias to threat. Am. J. Psychiatry 167, 694–698. doi: 10.1176/APPI.AJP.2009.09070956, 20395400

[ref4] Ben-Ezra M. Goodwin R. Leshem E. Hamama-Raz Y. (2023). PTSD symptoms among civilians being displaced inside and outside the Ukraine during the 2022 Russian invasion. Psychiatry Res. 320:115011. doi: 10.1016/j.psychres.2022.115011, 36566594

[ref5] Beri Drezner N. Tilbor E. Menkes-Caspi N. Krivoy A. Lurie I. (2024). The use of ambulatory psychiatric services among the Israeli population following the October 7 terror attacks and Iron swords war [Hebrew]. Harefuah 163, 484–487. 39114997

[ref6] Bitton S. Tuval-Mashiach R. Freedman S. (2017). Distress levels among parents of active duty soldiers during wartime. Front. Psychol. 8:1679. doi: 10.3389/fpsyg.2017.01679, 29018394 PMC5622972

[ref7] Bonanno G. A. Westphal M. Mancini A. D. (2011). Resilience to loss and potential trauma. Annu. Rev. Clin. Psychol. 7, 511–535. doi: 10.1146/ANNUREV-CLINPSY-032210-104526, 21091190

[ref8] Bryant R. A. Friedman M. J. Spiegel D. Ursano R. Strain J. (2011). A review of acute stress disorder in DSM-5. Depress. Anxiety 28, 802–817. doi: 10.1002/DA.20737, 21910186

[ref9] Casey L. Wright M.-A. Clough B. (2014). Comparison of perceived barriers and treatment preferences associated with internet-based and face-to-face psychological treatment of depression. Int. J. Cyber Behav. Psychol. Learn. 4, 16–22. doi: 10.4018/ijcbpl.2014100102

[ref10] Chorna N. (2024). Online psychological support amid the Russo-Ukrainian war: navigating mental health challenges. SSRN Electron. J. doi: 10.2139/ssrn.4747281

[ref11] da Silva H. C. Furtado da Rosa M. M. Berger W. Luz M. P. Mendlowicz M. Coutinho E. S. F. . (2019). PTSD in mental health outpatient settings: highly prevalent and under-recognized. Rev. Bras. Psiquiatr. 41, 213–217. doi: 10.1590/1516-4446-2017-0025, 30328959 PMC6794137

[ref12] Daniels S. Zalsman G. Itzhaky L. Szepsenwol O. Mahlev E. S. Benatov J. (2026). Suicidality calls to a national helpline: one year post the October 7 terror attack and amidst a prolonged war. J. Psychiatr. Res. 196, 18–23. doi: 10.1016/j.jpsychires.2026.02.003, 41650568

[ref13] Dominguez D. V. Cohen M. Brom D. (2004). Trauma and dissociation in psychiatric outpatients. Isr. J. Psychiatry Relat. Sci. 41, 98–110. 15478455

[ref14] Ferracioli N. G. M. Oliveira-Cardoso É. A. de Oliveira W. A. de Santos M. A. dos (2023) Potentialities and barriers of online psychotherapy during the COVID-19 pandemic: scoping review. Psicol. Teoria e Pesqui. 39:e22079 doi: 10.1590/0102.3772e39410.en

[ref15] Foa E. B. McLean C. P. Zang Y. Zhong J. Powers M. B. Kauffman B. Y. . (2016). Psychometric properties of the posttraumatic diagnostic scale for DSM-5 (PDS-5). Psychol. Assess. 28, 1166–1171. doi: 10.1037/PAS0000258, 26691504

[ref16] Greene T. Itzhaky L. Bronstein I. Solomon Z. (2018). Psychopathology, risk, and resilience under exposure to continuous traumatic stress: a systematic review of studies among adults living in southern Israel. Traumatology (Tallahass. Fla) 24, 83–103. doi: 10.1037/trm0000136

[ref17] Hamama L. Sarid O. Hamama-Raz Y. (2025). Psychological distress, resources, and coping strategies among evacuees and non-evacuees from an armed conflict zone: a network analysis. Stress. Health 41:e3525. doi: 10.1002/SMI.3525,, 39698890 PMC11656507

[ref18] Israel Central Bureau of Statistics. (2023a) Characterization and classification of geographical units by the socio-economic level of the population, 2019 (Publication No. 1903) [Data report]. Available online at: https://www.cbs.gov.il/en/publications/Pages/2023/socio-2019-e.aspx

[ref19] Israel Central Bureau of Statistics. (2023b) Peripherality index of localities and local authorities – 2020 (Publication No. 1917) [Data report]. Available online at: https://www.cbs.gov.il/en/publications/pages/2023/peripheriality-index-of-localities-and-local-authorities-2020.aspx

[ref20] Israel Home Front Command. (2025) How much time to reach the protected space? Available online at: https://www.oref.org.il/eng/articles/protection-guidelines/preparing-protected-space/1103

[ref21] Kaim A. Bodas M. (2024). The impact of 24/7 news coverage on the mental health of Israelis in the “Iron swords” war: a cross-sectional analysis among television audience. Stress. Health 40:e3398. doi: 10.1002/SMI.3398, 38544300

[ref22] Katsoty D. Greidinger M. Neria Y. Segev A. Lurie I. (2024). A prediction model of PTSD in the Israeli population in the aftermath of october 7th, 2023, terrorist attack and the Israel-Hamas war. Isr. J. Health Policy Res. 13:63. doi: 10.1186/S13584-024-00644-6, 39472957 PMC11520871

[ref23] Krivoy A. Rosenthal G. (2025). Wake-up call for recovery: a paradigm shift to address the deep crisis in Israel’s public mental health services in the shadow of October 7, 2023. Isr. J. Health Policy Res. 14:6. doi: 10.1186/s13584-025-00670-y, 39881384 PMC11776319

[ref24] Kroenke K. Spitzer R. L. Williams J. B. W. (2001). The PHQ-9: validity of a brief depression severity measure. J. Gen. Intern. Med. 16, 606–613. doi: 10.1046/J.1525-1497.2001.016009606.X, 11556941 PMC1495268

[ref25] Levi-Belz Y. Amsalem D. Groweiss Y. Blank C. Neria Y. (2025). The attack is not over yet: the impact of direct exposure to the October 7, 2023, attack on trajectories of PTSD and depression among the Israeli population. Psychol. Trauma 17, 1505–1513. doi: 10.1037/tra0001933, 40338540

[ref26] Levi-Belz Y. Groweiss Y. Blank C. Neria Y. (2024). PTSD, depression, and anxiety after the October 7, 2023 attack in Israel: a nationwide prospective study. EClinicalMedicine 68:102418. doi: 10.1016/j.eclinm.2023.102418, 38586476 PMC10994954

[ref27] Lu W. Yanos P. T. Waynor W. R. Gao C. E. Bazan C. Giacobbe G. . (2021). Trauma exposure and prolonged grief disorder among persons receiving community mental health services: rates and correlates. Front. Psych. 12:760837. doi: 10.3389/fpsyt.2021.760837, 35185633 PMC8854856

[ref28] Lurie I. Wolff-Sagy Y. Feldhamer I. Greidinger M. Yedidya R. Shmulevich M. . (2026). Resilience coaches: first year outcomes of a large-scale, low-intensity stratified intervention program in the public mental health system in Israel. Psychiatry Res. 355:116819. doi: 10.1016/j.psychres.2025.116819, 41273962

[ref29] Madoro D. Kerebih H. Habtamu Y. G/tsadik M. Mokona H. Molla A. . (2020). Post-traumatic stress disorder and associated factors among internally displaced people in South Ethiopia: a cross-sectional study. Neuropsychiatr. Dis. Treat. 16, 2317–2326. doi: 10.2147/NDT.S267307,, 33116530 PMC7548318

[ref30] Marciano H. Kimhi S. Eshel Y. Adini B. (2024). Resilience and coping during protracted conflict: a comparative analysis of general and evacuees populations. Isr. J. Health Policy Res. 13:56. doi: 10.1186/S13584-024-00642-8, 39358809 PMC11448295

[ref31] Matsumoto K. Hamatani S. Shimizu E. (2021). Effectiveness of videoconference-delivered cognitive behavioral therapy for adults with psychiatric disorders: systematic and Meta-analytic review. J. Med. Internet Res. 23:e31293. doi: 10.2196/31293, 34898445 PMC8713091

[ref32] National Insurance Institute of Israel. (2025) Help to anxiety victims. Available online at: https://www.btl.gov.il/English%20Homepage/Benefits/Benefits%20for%20Victims%20of%20Hostilities/Pages/Anxiety-Victims.aspx

[ref33] Neria Y. Besser A. Kiper D. Westphal M. (2010). A longitudinal study of posttraumatic stress disorder, depression, and generalized anxiety disorder in Israeli civilians exposed to war trauma. J. Trauma. Stress. 23, 322–330. doi: 10.1002/JTS.20522, 20564364

[ref34] Neria Y. Markowitz J. C. Amsalem D. Levi-Belz Y. Roe D. Lurie I. . (2025). Israeli mental health in the aftermath of the October 7 terrorist attack: risks, challenges, and recommendations. Isr. J. Health Policy Res. 14:25. doi: 10.1186/S13584-025-00682-8, 40240897 PMC12004549

[ref35] Peleg O. Gendelman L. (2025). Early evidence on the emotional distress of civilians, including evacuees, during a recent conflict. Int. J. Psychol. 60:e70048. doi: 10.1002/IJOP.70048, 40325823 PMC12053297

[ref36] Peterson A. L. Mintz J. Moring J. C. Straud C. L. Young-McCaughan S. McGeary C. A. . (2022). In-office, in-home, and telehealth cognitive processing therapy for posttraumatic stress disorder in veterans: a randomized clinical trial. BMC Psychiatry 22:41. doi: 10.1186/S12888-022-03699-4, 35038985 PMC8763446

[ref37] Rahamim O. Segev A. Sinai D. (2025). Benzodiazepine prescribing patterns following mass traumatic events. JAMA Psychiatry 82, 1133–1136. doi: 10.1001/jamapsychiatry.2025.1981, 40802247 PMC12351468

[ref38] Reuveni I. Tene O. Katz C. L. (2025). The personal and the national: lessons learned in the aftermath of the October 7 attacks in Israel. Psychiatry Res. 344:116332. doi: 10.1016/j.psychres.2024.116332, 39731883

[ref39] Roberts N. P. Kitchiner N. J. Kenardy J. Lewis C. E. Bisson J. I. (2019). Early psychological intervention following recent trauma: a systematic review and meta-analysis. Eur. J. Psychotraumatol. 10:1695486. doi: 10.1080/20008198.2019.1695486, 31853332 PMC6913678

[ref40] Sberro-Cohen S. E Ellen M. (2025). From conflict to care - telemedicine utilization during wartime: a retrospective cohort study. J. Med. Syst. 49:83. doi: 10.1007/s10916-025-02220-0, 40526202 PMC12174172

[ref41] Shalev L. Avni A. Tene O. Reuveni I. Avirame K. Eitan R. . (2024). Utilization of psychiatry services in the emergency department following a terror event in Israel. Psychiatry Res. 339:116059. doi: 10.1016/j.psychres.2024.116059, 38945102

[ref42] Shapira S. Sold A. Refaeli T. (2025). The interplay of personal and collective resilience and mental health during prolonged conflict: insights from young adults in Israel. Stress. Health 41:e70047. doi: 10.1002/SMI.70047,, 40347433 PMC12065528

[ref43] Sheerin C. Berenz E. C. Knudsen G. P. Reichborn-Kjennerud T. Kendler K. S. Aggen S. H. . (2016). A population-based study of help seeking and self-medication among trauma-exposed individuals. Psychol. Addict. Behav. 30, 771–777. doi: 10.1037/ADB0000185, 27269293 PMC5114150

[ref44] Sijbrandij M. Kunovski I. Cuijpers P. (2016). Effectiveness of internet-delivered cognitive behavioral therapy for posttraumatic stress disorder: a systematic review and META-analysis. Depress. Anxiety 33, 783–791. doi: 10.1002/DA.22533, 27322710

[ref45] Smulson M. Nazar M. Meshcheriakov D. Ditjuk P. Zinchenko O. Starkov D. (2025). Role of the virtual space in psychological support of adult Ukrainian civilians during wartime. Int. J. Educ. Inf. Technol. 19, 1–9. doi: 10.46300/9109.2025.19.1

[ref46] Spitzer R. L. Kroenke K. Williams J. B. W. Löwe B. (2006). A brief measure for assessing generalized anxiety disorder: the GAD-7. Arch. Intern. Med. 166, 1092–1097. doi: 10.1001/ARCHINTE.166.10.1092, 16717171

[ref47] State Comptroller and Ombudsman of Israel. (2024) Mental health care following the events of October 7, 2023, and the Iron Swords War. State Comptroller and Ombudsman Office [in Hebrew]. Available online at: https://chrome-extension://efaidnbmnnnibpcajpcglclefindmkaj/https://library.mevaker.gov.il/sites/DigitalLibrary/Documents/2025/Swords-of-Iron-1/EN/2025-Swords-of-Iron-100-Taktzir-EN.pdf

[ref48] Wittmann L. Dimitrijevic A. Ehlers A. Foa E. B. Kessler H. Schellong J. . (2021). Psychometric properties and validity of the German version of the post-traumatic diagnostic scale for DSM-5 (PDS-5). Eur. J. Psychotraumatol. 12:1965339. doi: 10.1080/20008198.2021.1965339, 34589176 PMC8475123

[ref49] Zandieh S. Abdollahzadeh S. M. Sadeghirad B. Wang L. McCabe R. E. Yao L. . (2024). Therapist-guided remote versus in-person cognitive behavioural therapy: a systematic review and meta-analysis of randomized controlled trials. Can. Med. Assoc. J. 196, E327–E340. doi: 10.1503/CMAJ.230274, 38499303 PMC10948182

[ref50] Zerach G. (2024). Deployment stressors, mental health outcomes, and protective factors among wives of reserve soldiers during the Israel-Hamas war: a latent profile analysis approach. Stress. Health 40:e3497. doi: 10.1002/smi.3497, 39485706

[ref51] Zhu X.-L. Wen Z. Yu W.-B. (2023). Effects of media exposure on PTSD symptoms in college students during the COVID-19 outbreak. Front. Public Health 11:1050759. doi: 10.3389/fpubh.2023.1050759, 37228721 PMC10203595

